# Who is Responsible for Responsible Innovation? Lessons From an Investigation into Responsible Innovation in Health: Comment on "What Health System Challenges Should Responsible Innovation in Health Address? Insights From an International Scoping Review"

**DOI:** 10.15171/ijhpm.2019.32

**Published:** 2019-05-19

**Authors:** Bernd Carsten Stahl

**Affiliations:** De Montfort University, Leicester, UK.

**Keywords:** Responsible Innovation, Responsible Research and Innovation, Responsible Innovation in Health

## Abstract

Responsible innovation in health (RIH) takes the ideas of responsible research and innovation (RRI) and applies them to the health sector. This comment takes its point of departure from Lehoux et al which describes a structured literature review to determine the system-level challenges that health systems in countries at different levels of human development face. This approach offers interesting insights from the perspective of RRI, but it also raises the question whether and how RRI can be steered and achieved across healthcare systems. This includes the question who, if anybody, is responsible for responsible innovation and which insights can be drawn from the systemic nature RIH.

Responsible Innovation in Health (RIH) is a concept put forward by Lehoux et al.^1^ It draws on the discourse surrounding responsible innovation^2^ or responsible research and innovation (RRI).^3^ The idea behind these concepts is to ensure that research and innovation activities are acceptable, desirable and sustainable. RIH takes these ideas and transfers them to the health sector. In their paper Lehoux et al^1^ present the findings from a structured literature review that aimed to identify system-level challenges that health systems currently face. The idea motivating this research is that an understanding of the key challenges facing a health system can guide innovations and innovation policy, to ensure that their consequences promote the public good and that the overall innovation process is thereby responsible.

This approach is interesting from the point of view of responsible innovation in that it attempts to answer a question which is often not addressed, namely the question of the intended and desirable outcomes that motivate an innovator. Focusing on system-level challenges sidesteps the question of which ethical or other values need to motivate responsible innovation to render it responsible. The approach has the advantage of offering the potential of a practical goal to be pursued. By focusing on systems-level challenges, the paper moves beyond the focus on the individual researcher, research lab or institute that much of the literature on responsible innovation focuses on and thereby highlights a key aspect of responsible innovation that is often under investigated.

The paper is furthermore interesting in that it splits these system-level challenges of health systems in accordance with the level of development of the countries in question. It is plausible that health systems in richer countries have different priorities or problems and require different approaches from those in poorer countries. Lehoux et al^1^ demonstrate that this intuition is supported by evidence from the literature.

When looking at the system-level challenges presented in the paper, it becomes clear, however, that this high-level view is not sufficient to really grasp the problems that healthcare systems face in practice. The aggregate data presented in the paper remains too abstract to provide practical insights. For example, the categories of challenges that are most pressing in all four groups of countries with different levels of development are in all cases health service delivery, human resources or leadership and governance. There are differences in ranking these between different groups of countries, with health service delivery being a more pronounced problem in countries with a higher human development index, whereas human resources is seen as the number one problem in countries with lower scores on the higher human development index. However, I would assume that human resources challenges will look very different in different health systems, but in order to understand what the challenges in fact are, one would have to go back to the original publications that were analysed for the review. The approach can therefore be said to be interesting in that it proposes new ways of thinking about RIH and the emphasis that research and innovation policy should pursue, but it does not move far enough to allow deducing practical suggestions.

Having said this, the paper is still an interesting contribution to the debate around responsible innovation. One aspect that I found particularly stimulating is the fact that the paper raises the question of the relationship between innovations and the high-level aims which such innovation is meant to achieve. The reason why this question is implicitly posed by the paper is that the analysis of the system-level challenges and individual innovation activities becomes very difficult to establish. The system-level challenges are mostly issues of the broader context of innovation and directly related to the socio-economic constitution of the society that they find themselves.

Challenges that are discussed in the paper include frugal innovation, ie, low-cost, local- or community-based innovation that deals with financial pressure on health system and contribute to affordability of health care. Challenges related to human resources can be translated into requirements for innovation to be easy to use and thereby require less training. Another key challenge is that of the influence of economic incentives on the governance of health systems which is closely related to regulatory frameworks and market dynamics. Questions of infrastructure capacity and distribution systems form another group of important challenges across various types of health systems. A final set of issues is related to knowledge and knowledge management, in particular the use of IT-based solutions and services.

What this list of key challenges demonstrates is that the main issues that systems have to contend with are not so much based on available technologies, drugs or facilities but on the socio-economic make-up of the healthcare system itself. Problems here have to do with distribution of resources, principles of distribution of access and are fundamentally related to the equitable treatment of different groups and demographics. Most of these issues are broadly independent of research and innovation activity. In fact, I would argue that the impact that the majority of health-related innovations have on the larger scale healthcare systems is largely undetermined. To put it differently, whether a new piece of information technology saves money and makes healthcare more available or costs more money and reduces the availability of healthcare provision is not a function of the innovation itself, but of the environment in which it is put to use. This argument is informed by a long tradition of research that argues for the co-construction of science, technology and society. It is difficult to impossible to separate the innovation itself from the environment in which it is used. Technologies are always designed for specific environments and users, whether intentionally or unintentionally.^4^ At the same time, the logical malleabitlity in particular of information and communication technologies^5^ means that their degree of interpretive flexibility,^6^ ie, the difficulty of predicting their eventual use and its consequences is even higher than it is for other technologies. The problem with the high level review undertaken by Lehoux et al^1^ is that this context specificity of the technology is in danger of becoming invisible and the technology can be interpreted as context independent.

This raises interesting questions around the possibility and usefulness of RIH and the distribution of responsibilities that would render RIH effective. It would mean that the question whether a particular innovation is considered as an expression of responsibility is largely independent of the process of innovation as well as its eventual outputs. The nature of the innovation as being responsible would be determined by the eventual use that it is put to in a particular healthcare system. This, in turn, has the interesting side effect that the place of responsibility is no longer located with the individual researcher, research group or institution but resides on a higher level in the healthcare system. Such a conclusion would go counter to the majority of literature in RRI which tends to focus on responsibilities of the researcher or the organisation that this undertaking the research.

This situation thus begs the question posed in the title of this paper: who is actually responsible for responsible innovation? If responsible innovation is about ensuring the desirability, acceptability and sustainability of research and innovation processes and products, then who is in a position to influence those and steer them in a desirable direction? Much of the literature on responsible innovation focuses on the responsibilities of researchers or research institutions. One interpretation of the Lehoux et al^1^ paper could be to reject this focus and explore whether and to what degree other stakeholders, groups or institutions can be seen as responsible for the outcomes of health innovation.

I will not pretend that I have an easy answer to the question of who is responsible for health innovation. However, it would seem to reinforce the importance of reconsidering the very notion of responsible innovation. I have argued elsewhere that a useful reading of the idea of responsible innovation is that of a “higher-level or meta-responsibility that aims to shape, maintain, develop, coordinate and align existing and novel research and innovation-related processes, actors and responsibilities with a view to ensuring desirable and acceptable research outcomes.”^7^ This idea is based on the recognition that the responsibilities that each human being has are complex, overlapping and interwoven. They are better understood as overlapping networks than as simple linear attributions.^8,9^ These suggestions are based on the insight that technologies are products of the social environment of their creation and use that can be found in the various streams of literature that inform the discourse of RRI, such as philosophy of technology,^10^ computer and information ethics,^11^ technology assessment^12^ or science and technology studies.^13,14^

What this means for RIH is that a first requirement for responsible innovation is a better understanding of the existing networks of responsibility in a particular healthcare system. This will require difficult investigations into complex political processes, for example with regards to how incentives are defined and determined which pathways exist from research funding to the introduction of innovative technologies or services in a healthcare setting. The outcome of a particular innovation as meeting the system-level challenges and thus being counted as responsible, will depend on the interplay of a potentially large number of individuals and institutions. The eventual success or lack thereof is largely outside of the control of any one individual and notably beyond what could be expected from an individual researcher or innovator.

This then brings us back to the question formulated in the title: who is responsible for responsible innovation? The answer seems to be elusive there is no individual, group or institution that carries responsibility for responsible innovation. This may lead to several different possible conclusions: One could argue that there is nobody who is responsible for responsible innovation and that, at best, it is an unpredictable outcome of a systemic interplay of numerous agents.

A different conclusion might be that following the idea of responsible innovation requires rethinking the entire research and innovation ecosystem with a view to ensuring that innovation processes and outcomes meet societal goals. This would probably require a messy intervention into the research and innovation policy system, which will need the input from all stakeholder groups, including patients, researchers, head providers, policy-makers and others. What exactly such an intervention might look like is difficult to predict and is also not clear whether it would be successful. However, if we do not go down this route then the conclusion may be that there is nobody who is responsible for responsible innovation and it is not clear to me whether it then remains meaningful and useful to speak of responsible innovation or RIH. The conclusion one could therefore draw from Lehoux et al’s paper is that it calls for an entire research programme to understand the research and innovation life cycle and ecosystem to determine how the system-level outcomes described in the paper can be achieved and responsible innovation can be realised. Such research has already begun (see, for example, Williams and Woodson^15^) but will require significant efforts to come to fruition.

## Ethical issues

Not applicable.

## Competing interests

Author declares that he has no competing interests.

## Author’s contribution

BCS is the single author of the paper.
